# Google Trends Assessment of Keywords Related to Smoking and Smoking Cessation During the COVID-19 Pandemic in 4 European Countries: Retrospective Analysis

**DOI:** 10.2196/57718

**Published:** 2024-12-03

**Authors:** Tobias Jagomast, Jule Finck, Imke Tangemann-Münstedt, Katharina Auth, Daniel Drömann, Klaas F Franzen

**Affiliations:** 1 Medical Clinic III University of Lübeck Lübeck Germany; 2 Airway Research Center North Deutsches Zentrum für Lungenforschung Großhansdorf Germany

**Keywords:** internet, coronavirus, COVID-19, SARS-CoV-2, pandemics, public health, smoking cessation, tobacco products, Google Trends, relative search volume, Europe, online, search, smoking, addiction, quit, cessation, trend, cluster, public interest, lockdown, vaccination, spread, incidence

## Abstract

**Background:**

Smoking is a modifiable risk factor for SARS-CoV-2 infection. Evidence of smoking behavior during the pandemic is ambiguous. Most investigations report an increase in smoking. In this context, Google Trends data monitor real-time public information–seeking behavior and are therefore useful to characterize smoking-related interest over the trajectory of the pandemic.

**Objective:**

This study aimed to use Google Trends data to evaluate the effect of the pandemic on public interest in smoking-related topics with a focus on lockdowns, vaccination campaigns, and incidence.

**Methods:**

The weekly relative search volume was retrieved from Google Trends for England, Germany, Italy, and Spain from December 31, 2017, to April 18, 2021. Data were collected for keywords concerning consumption, cessation, and treatment. The relative search volume before and during the pandemic was compared, and general trends were evaluated using the Wilcoxon rank-sum test. Short-term changes and hereby temporal clusters linked to lockdowns or vaccination campaigns were addressed by the flexible spatial scan statistics proposed by Takahashi and colleagues. Subsequently, the numbers of clusters after the onset of the pandemic were compared by chi-square test.

**Results:**

Country-wise minor differences were observed while 3 overarching trends prevailed. First, regarding cessation, the statistical comparison revealed a significant decline in interest for 58% (7/12) of related keywords, and fewer clusters were present during the pandemic. Second, concerning consumption, significantly reduced relative search volume was observed for 58% (7/12) of keywords, while treatment-related keywords exhibited heterogeneous trends. Third, substantial clusters of increased interest were sparsely linked to lockdowns, vaccination campaigns, or incidence.

**Conclusions:**

This study reports a substantial decline in overall relative search volume and clusters for cessation interest. These results underline the importance of intensifying cessation aid during times of crisis. Lockdowns, vaccination, and incidence had less impact on information-seeking behavior. Other public measures that positively affect smoking behavior remain to be determined.

## Introduction

### COVID-19 Pandemic in Europe

The SARS-CoV-2 (COVID-19) pandemic posed unprecedented challenges to global health care and public health. SARS-CoV-2, the virus responsible for COVID-19 infection, initially surfaced in Wuhan, China, in December 2019 and quickly spread worldwide [1]. The first cases in Europe were reported in January 2020, shortly after it was declared a global pandemic by the World Health Organization (WHO) [2].

### Smoking is a COVID-19 Risk Factor

European countries exhibited disparate patterns of incidence and mortality rates during the pandemic that triggered national governments to pass varying public restrictions, for example, the shutdown of public institutions, curfews, quarantine, and the use of face masks in public spaces [3,4]. The predominant manifestation of COVID-19 infections is characterized by the clinical triad of cough, fever, and symptoms resembling those of influenza [5]. In the initial stages of the pandemic, there ensued a discourse regarding putative protective attributes associated with smoking, as a study showed a lower incidence of COVID-19 infection among smokers [6]. These studies received a lot of media attention. However, as cumulative data have been assimilated, smoking is currently discerned as a substantial risk factor for potentially life-threatening consequences following infection [5,7-11]. Also, the authors of the mentioned studies had severe nondeclared conflicts of interest with the tobacco industry [12]. Hence, in light of the ongoing COVID-19 pandemic, smoking as a modifiable risk factor has re-emerged as a focal point within public health concerns.

### Changes in Smoking Behavior During the Pandemic

Many studies addressed smoking behavior during the COVID-19 pandemic in different locations, during diverging periods, and using varying methods [13-19]. A large meta-analysis points to an increase in smoking [15]. Still, no general conclusion can be drawn from the presented evidence regarding changes in consumption and cessation. Smoking cessation can be categorized into 2 groups. One is commercially available substitutes, such as nicotine patches and e-cigarettes. The latter are advertised as less harmful cessation aids. Despite that, the current evidence is ambiguous and their role during the COVID-19 pandemic is understudied [17]. In addition, expert guidance is available for smoking cessation. Yet during the pandemic and due to associated restrictions regarding mobility and face-to-face meetings, availability might have been a challenge for patients [20]. In a previous study, our group shed light on the quitting behavior of smokers and ex-smokers in England, Germany, Spain, and Italy during the pandemic [21]. Despite psychological distress about the severe outcomes of COVID-19 infection, no higher rates of smoking cessation attempts were observed. Individuals may react both ways and augment their consumption patterns as a mechanism for stress mitigation or demonstrate an increased interest in cessation programs or the adoption of alternative nicotine replacement products due to a fear of severe outcomes [21-24].

### Methodological Introduction to Google Trends Data

This follow-up study further investigates the aforementioned countries. Google Trends data were retrieved to monitor public interest in smoking, cessation, and treatment. Google Trends data reflect interest for keywords on a scale from 0 to 100 (relative search volume [RSV]) relative to all search inquiries in a given period and location. Google Trends data have been proven a valid tool in medical research and have been applied throughout various fields of medicine, for example, evaluating the effects of interventions or forecasting future trends to prepare health care providers [25,26]. In smoking-related research, different tobacco control measures have been evaluated using RSV [27-30]. Furthermore, a study by Cavazos-Rehg et al [31] linked the information-seeking behavior on Google with real-life use of tobacco products. Google Trends data have the advantage of being easily accessible, objective, and always up-to-date. These properties are especially useful in the dynamic event of a pandemic [32,33]. Furthermore, during the COVID-19 pandemic, people turned to the internet for guidance on health-related topics [34]. Previous publications displayed an increase in RSV for selected mental health–related keywords [32,35]. As cited above, psychological distress also seems to play a major role in motivation for cessation [21-23]. In line, studies at the intersection of Google Trends data and smoking research during the pandemic are limited but showed stable or decreased interest in cessation [16,19,36].

### Hypothesis, Study Design, and Implications

Based on previous research, we hypothesized that major events during the pandemic caused substantial changes in public interest for smoking-related topics concerning consumption, cessation, and treatment [15,16,36]. Among these major events, it was assumed that lockdowns, vaccination campaigns, and rising incidence would induce the greatest echo in Google Trends data [33]. From the results of available survey studies, we expected the RSV to mirror the trend of increased interest in consumption and a decrease in cessation and treatment [15].

For investigation, a reasonable period was selected from December 31, 2017, to April 4, 2021, capturing the dynamics of the transition into the pandemic state and comprising multiple waves of infection. Changes in RSV were evaluated in 3 separate ways. First, the overall RSV for the abovementioned domains after the start of the pandemic were compared. Second, short-term changes in RSV were addressed. Cluster analysis was conducted to discern the lockdown measures, the start of vaccination, and changing incidence rates that evoked the greatest levels of interest, in terms of RSV. Third, the general occurrence of clusters was compared between the before and during the pandemic state.

Identification of optimal timing and kind of intervention is essential to protect vulnerable groups during a public health crisis. Currently, evidence in the field of smoking cessation during the pandemic is mainly reliant on survey-generated data and is heterogeneous. RSV data are a cost-effective and instantaneous tool to monitor public interest and might effectively supplement the survey data. By screening various keywords, appropriate interventions might be determined. These insights could guide policy makers and health care providers: first, if intensification of public campaigns for cessation aid and information about treatment is necessary in times of pandemics and second, to follow up on possible detrimental effects on smoking behavior due to political measurements such as lockdowns.

## Methods

### Data Collection

Google Trends query was carried out based on methodological suggestions by Nuti et al [25]. The weekly RSV was retrieved from Google Trends on July 1, 2023, for each country during the period from December 31, 2017, to April 18, 2021, to investigate a reasonable time preceding the pandemic to identify changes [37]. The preceding period had to be long enough to serve as a comparison mirroring long-term trends and patterns. The observed period after the onset of the pandemic spanned approximately the first 3 waves of infections according to the Robert Koch Institute and hereby sufficiently reflects dynamics at the beginning of the pandemic [38]. Major events during the pandemic were lockdowns and vaccinations. The dates of these events are country-wise depicted in Table 1. Data were collected for the indicated keywords, as listed in Table 2. All query categories were searched, no quotation marks were used, and locations were set according to the 4 countries. To examine alterations in the search behavior surrounding smoking-related keywords, our study targeted 3 specific domains: cessation, treatment, and consumption. A comprehensive translation of the relevant keywords into all 4 designated languages was conducted. Word selection was carried out based on expert consensus and literature review. Most common tobacco products and treatment options were filtered [39-44]. For terms regarding cessation, previous research concerning RSV data in the context of cessation was searched [16,28-30,36]. However, choices were restricted by the overlapping availability of Google Trends data for each country. Otherwise, keywords were chosen to display a broad spectrum of each abovementioned domain. Noteworthy Champix (trade name for varenicline) was chosen over varenicline as varenicline showed higher variability in RSV over time, increasing the susceptibility of this keyword to outliers. In the following text, the English term is used for cases of subsumption.

**Table 1:** Dates of major events by country. Since there is high heterogeneity of lockdown measures between the investigated countries, the listing of specific bans was omitted. However, shared characteristics of lockdowns were the closure of public cultural facilities, curfew, the ban on assembly, and restriction of mobility.

	England	Germany	Italy	Spain
Start of lockdowns	March 23, 2020 November 5, 2020 January 6, 2021	March 22, 2020 November 1, 2020	March 10, 2020 October 26, 2020 March 15, 2021	March 14, 2020 October 25, 2020
Start of vaccination	December 8, 2020	December 27, 2020	December 27, 2020	December 27, 2020

**Table 2:** Keywords by country and domain.

Domain	England	Germany	Italy	Spain
Consumption	Cigarette Cigar Tobacco	Zigarette Zigarre Tabak	Sigaretta Sigaro Tabacco	Cigarrillo Cigaro Tabaco
Cessation	Smoking cessation Smoke free Stop smoking	Raucherentwöhnung Rauchfrei Rauchen aufhören	Smettere di fumare Anti fumo Non fumatore	Deshabituatión tabaquita Libre de humo Dejar de fumar
Treatment	Nicotine patch e-cigarette Champix	Nikotinpflaster E-Zigarette Champix	Cerotti alla nicotina Sigaretta electtronica Champix	Parche de nicotina Cigarillo electrónico Champix

RSV serves as a temporally resolved representation of search behavior concerning a designated keyword. The term “relative” refers to the quantitative assessment of search queries associated with the keyword relative to the total search query volume prevalent at a given point in time in a specified location. To facilitate meaningful comparisons and emphasize variations in search term popularity over time, the time point at which this ratio reaches its maximum is conventionally designated as having an RSV of 100. All other values within the examined period are subsequently expressed regarding this maximum.

Data concerning the COVID-19 pandemic incidence were retrieved from the UK Health Security Agency data dashboard for England [45] and from Our World in Data COVID-19 Data set for Germany, Italy, and Spain [4]. Lockdown measurements and the start of vaccination were selected and gathered from the media.

### Statistical Analysis

For statistical analysis of time series, 3 different approaches were used. First, for overall trends, the Wilcoxon rank-sum test was applied to compare the entire RSV by keywords before and during the pandemic. A similar approach was previously presented by Cunningham et al [36]. The onset was defined as January 24, 2020, with the first reported cases in Europe [2]. To verify that the date chosen for the division of the time series was appropriate, we confirmed by checking the RSV of common search terms linked to the pandemic (exemplary for Germany: “COVID-19,” “Coronavirus,” and “Pandemie”). The week of the first reported case in Europe coincided with the emerging interest in the indicated terms as RSV started to rise (Multimedia Appendix 1). These dynamics were also presented by Effenberger et al [46]. *P* values were adjusted using the Benjamini-Hochberg procedure to control for false discovery rate [47]. This approach only compares RSV before and during the pandemic, therefore fluctuations within the pandemic are beyond the scope of this kind of analysis. A higher temporal resolution is necessary to reveal changes in RSV within the time of the pandemic.

Hence, for short-term changes in RSV possibly linked to COVID-19–related events, a cluster detection test was used. This flexible scan statistics was introduced by Takahashi et al [48] and is a common approach in epidemiologic research and has been previously used in the context of RSV data [27]. For events evoking the greatest interest in a population, we anticipated lockdown measurements, vaccination campaigns, and steep rises in incidence, based on expert consensus and previous research [33]. R programming language (R Core Team) was used to apply the *FleXScan* package [49]. The settings for the algorithm were adapted from Tabuchi et al [27] and are (1) the prespecified significance level for the restriction α1=.2, (2) the significance level of the test α=.05, (3) replications of the Monte Carlo hypothesis testing 999, (4) maximum length of a cluster 17 weeks, and (5) minimum length of a cluster 2 weeks. For the baseline of expected RSV at an indicated date, the median RSV of the period 26 weeks flanking the corresponding date was used. This ensured that temporal clusters could be identified irrespective of long-term trends.

Third, a chi-square test was performed to compare the number of weeks that were part of clusters before and during the pandemic. Clusters were aggregated by domains. This was done to verify if the accumulation of periods with heightened interest was randomly distributed before and after the onset of the pandemic. *P*<.05 were considered statistically significant. Tabular data were handled in .xlsx format. R Studio and R programming language were used for all calculations and generation of plots [50,51].

### Ethical Considerations

The use of publicly available data and nonpersonally identifiable information within this research obviates the necessity for an ethics review process.

## Results

### Visual Inspection

**Figure 1:**
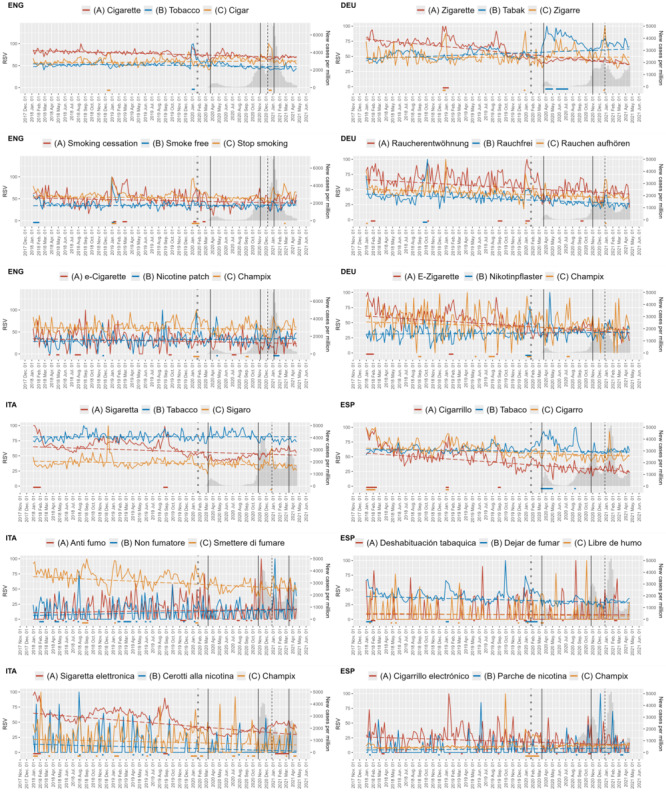
Relative search volume data and new cases per million over time. Dashed lines illustrate long-term trends in relative search volume calculated as linear regression. The vertical dotted line indicates the defined start of the pandemic in Europe with the first confirmed case on January 24, 2020. The vertical solid lines mark the start of lockdowns. The gray dashed lines mark the start of the vaccination campaigns. The color-coded solid lines below the x-axis mark the clusters. Countries are separated by quadrants: upper-left England (ENG), upper-right quadrant Germany (DEU), lower-left Italy (ITA), and lower-right Spain (ESP). RSV: relative search volume.

RSV for keywords of smoking consumption, cessation, and treatment from December 31, 2017, to April 4, 2021, are depicted in Figure 1. Visual inspection revealed 3 aspects. First, there are large differences in the variability of the RSV for the keywords examined. High variability is characterized by trends on the x-axis and isolated spikes with high RSV. Keywords in the consumption group appeared to be less variable, while terms in the cessation and treatment groups showed higher variability, especially in Italy and Spain (Figure 1). Second, overarching trend lines seem to decline within all domains and countries, with few exceptions that are later presented. Third, there seems to be no accumulation of substantial clusters after the start of the pandemic, again with few exceptions (Figure 1).

### Consumption

For the general effects of the pandemic, a comparison of the entire RSV was conducted before and after the start. Hereafter, a short-term relationship between lockdown measures and the start of vaccination and RSV data was established. Observations were made to ascertain whether clusters initiate or exhibit an abrupt termination with the commencement of lockdown measures.

For consumption, we saw an overarching trend of decline in RSV after the start of the pandemic. For 7 (58%) out of 12 consumption-related keywords, a significantly reduced RSV was observed. However, there were exceptions. “Cigar” had more RSV during the pandemic than before in England (*P*=.04). Besides, in Germany and Spain, we saw an increase in informational demand for “tobacco” (*P*<.001 and *P*=.03; Table 3). In short-term dynamics, long-lasting clusters for “tobacco” at the beginning of the pandemic for the aforementioned countries were observed. A second “tabaco” cluster emerged in Spain at the beginning of the second wave. Around the time of the start of the vaccination campaign, short-lasting clusters for cigar were observed in all countries except Spain (Figure 1). Overall, clusters regarding consumption during the pandemic only occurred significantly more frequently in Germany (*P*=.01). Otherwise, numbers remained stable (Table 4).

**Table 3:** Comparison of relative search volume before and during the COVID-19 pandemic using the Wilcoxon rank-sum test. *P* values were adjusted using the Benjamini-Hochberg procedure.

		Before the COVID-19 pandemic, median (IQR)	During the COVID-19 pandemic, median (IQR)	Trend during pandemic	Adjusted *P* value
**England**					
	Cigarette	80 (76-83)	71 (68-74)	↓^a^	<.001
	Tobacco	52 (50-56)	47 (44-53)	↓	<.001
	Cigar	59 (54-65)	63 (55-69)	↑^b^	.04
	Smoking cessation	51 (43-62)	45 (34-54)	↓	<.001
	Smoke free	37 (33-41)	35 (31-38)	↓	.03
	Stop smoking	57 (52-62)	55 (50-60)	=^c^	.16
	e-Cigarette	36 (23-47)	28 (17-37)	↓	<.01
	Nicotine patch	31 (22-38)	38 (28-43)	↑	.01
	Champix	61 (52-71)	60 (53-68)	=	.62
**Germany**					
	Zigarette	70 (65-75)	43 (41-45)	↓	<.001
	Tabak	51 (46-58)	72 (64-81)	↑	<.001
	Zigarre	48 (41-56)	50 (42-60)	=	.40
	Raucherentwöhnung	66 (57-78)	48 (42-57)	↓	<.001
	Rauchfrei	38 (34-43)	27 (25-31)	↓	<.001
	Rauchen aufhören	46 (42-53)	39 (36-43)	↓	<.001
	E-Zigarette	59 (48-67)	36 (33-41)	↓	<.001
	Nikotinpflaster	33 (24-38)	34 (29-41)	=	.15
	Champix	51 (38-67)	47 (37-55)	=	.07
**Italy**					
	Sigaretta	67 (61-73)	51 (45-57)	↓	<.001
	Tabacco	81 (77-85)	81 (77-86)	=	.95
	Sigaro	38 (35-42)	35 (30-40)	↓	<.01
	Anti fumo	18 (0-27)	12 (0-21)	=	.31
	Non fumatore	0 (0-24)	14 (0-21)	=	.18
	Smettere di fumare	71 (62-79)	53 (48-60)	↓	<.001
	Sigaretta elettronica	61 (54-68)	38 (32-43)	↓	<.001
	Cerotti alla nicotina	0 (0-0)	0 (0-0)	=	.08
	Champix	25 (0-37)	13 (0-29)	↓	.01
**Spain**					
	Cigarrillo	52 (41-62)	29 (24-35)	↓	<.001
	Tabaco	61 (59-65)	64 (59-72)	↑	.03
	Cigarro	67 (59-73)	52 (47-56)	↓	<.001
	Deshabituación tabaquica	0 (0-0)	0 (0-0)	=	.65
	Dejar de fumar	39 (33-44)	31 (27-34)	↓	<.001
	Libre de humo	0 (0-22)	0 (0-0)	=	.52
	Cigarrillo electrónico	25 (14-33)	15 (0-25)	↓	<.001
	Parche de nicotina	0 (0-0)	0 (0-0)	=	.63
	Champix	8 (7-11)	12 (10-14)	↑	<.001

^a^↓: significant decline.

^b^↑: significant incline.

^c^=: no significant changes corrected.

**Table 4:** Comparison of cluster weeks before and during the COVID-19 pandemic by chi-square test.

Country and period			Consumption							Cessation									Treatment					
			Cluster		No cluster	*P* value			Cluster			No cluster			*P* value		Cluster				No cluster			*P* value
**England**							>.99								.04									.81
	Before the COVID-19 pandemic	6		321				29			298					24				303				
	During the COVID-19 pandemic	3		189				7			185					16				176				
**Germany**							.01								.07									.01
	Before the COVID-19 pandemic	10		317				22			305					29				298				
	During the COVID-19 pandemic	17		175				5			187					5				187				
**Italy**							.24								<.001									.82
	Before the COVID-19 pandemic	10		317				53			274					44				283				
	During the COVID-19 pandemic	2		190				10			182					28				164				
**Spain**							.60								.22									.08
	Before the COVID-19 pandemic	24		303				36			291					19				308				
	During the COVID-19 pandemic	11		181				14			178					20				172				

### Cessation

As for consumption, a decrease in interest in cessation was noticeable across all countries for nearly all keywords, with 7 (58%) out of 12 keywords showing a significant decline. Keywords with nonsignificant changes were frequently affected by high variability in RSV, for example, “libre de humo” (*P*=.52) or “non fumatore” (*P*=.18)*.* No keyword showed significantly increased RSV during the pandemic (Table 3). In temporal evaluation, no relation between the occurrence of clusters and incidence, lockdowns, or vaccination could be established. Interestingly, England, Italy, and Spain showed an accumulation of clusters around the new year before the pandemic. Such clusters were missed in these countries during the pandemic (Figure 1). Generally, clusters for cessation were less frequent during the pandemic in all countries, especially in England and Italy, and significant differences were observed (*P*=.04 and *P*<.001; Table 4).

### Treatment

Treatment showed a heterogeneous picture. The comparison of the RSV did not yield uniform results. The only consistent finding across all countries was a decline in interest in “e-cigarette” (England *P*<.01; Germany, Italy, and Spain *P*<.001). Interest in “Champix” has risen in Spain (*P*<.001), whereas it has fallen in Italy (*P*<.01; Table 3). The cluster analysis revealed a long cluster for “Champix” in Spain, which began before the pandemic. Otherwise, there was a longer cluster for Italy concerning “sigaretta elettronica,” occurring between the second and third waves and ending with the start of the third lockdown. No other temporal correlations could be established (Figure 1). During the pandemic, only Germany showed a significantly shorter duration of clusters for treatment (*P*=.01). Here, similar to the effects noticed for cessation, before the pandemic, clusters of treatment around the turn of the year were present. These were absent during the pandemic (Table 4).

Generally, across all countries, the collected data point to less interest in the domain cessation during the COVID-19 pandemic. But also, the domain consumption was overall of less interest while the number of clusters remained mostly the same. Treatment-related keywords behaved less uniformly. Here, only e-cigarettes showed a country-spanning decline.

## Discussion

### Principal Findings

In this study, the effect of the COVID-19 pandemic on internet search query data concerning smoking-related keywords was investigated. Despite some differences in country-specific manner, overarching trends are displayed. This study comprises 3 main findings. First, through analysis of overall RSV before and during the pandemic, a substantial drop in interest in the domains of cessation, and second, consumption was observed. Treatment showed heterogeneous trends in a country-dependent manner. Third, in short-term analysis, a sparse relationship of substantial clusters to lockdown measurements or the start of vaccination campaigns could be established as discussed in the following paragraphs concerning consumption, cessation, and treatment. These events seem to have little influence on search queries. Clusters are preferably evoked by other incidents. However, as seen in the overall RSV comparison, a trend toward lower interest in cessation during the pandemic was presented with fewer cluster weeks. For consumption, the number of cluster weeks remained stable during the pandemic despite a decrease in overall RSV for this domain, possibly pointing to events beyond the scope of this study triggering spikes of interest despite a decline in overall interest.

Research on the interplay between the COVID-19 pandemic and smoking exhibits multifaceted outcomes. Initially, smokers may have perceived smoking cessation as unnecessary due to emerging reports suggesting nicotine’s potential protective role against severe COVID-19 infection [6,13,52]. However, in the short term, mounting evidence underscored the deleterious impact of smoking on the disease trajectory [7-9,11]. Intuitively, one might hypothesize that this phenomenon prompted numerous smokers to limit their smoking habits, albeit temporarily [53]. However, diverging from this expectation, the majority of empirical studies conducted during the pandemic across diverse global regions present contradictory findings. The meta-analysis conducted by Bakaloudi et al [15] elucidates a prevailing pattern of escalated or maintained smoking behavior across most studies. A plausible explanation for the observed phenomenon is the characterization of the COVID-19 pandemic as a stressor, rendering smoking cessation more challenging [54]. Research demonstrating a positive correlation between elevated stress levels and smoking behavior is noteworthy. [14,22,55,56]. Smoking is construed as a coping mechanism to manage psychosocial stressors associated with lockdown scenarios [23,24,57-59].

### Consumption

As opposed to the study by Bakaloudi et al [15] mentioned in the *Introduction* section that investigated worldwide trends, our investigation revealed a decline in consumption-associated RSV. There might be two possible explanations: (1) there is a plethora of studies that account for regional differences and (2) some also report a decline in smoking during the pandemic [23,53]. In Spain, smokers decreasing their consumption seem to outnumber users with increased consumption [60]. Interestingly, the same study reports a decline in sales for cigarettes and cigars during the pandemic with a substantial rise in tobacco sales that resamples our findings from RSV analysis. Besides, before the onset of the pandemic, a discernible trend emerged across the European Union marked by a general decrease in smokers [61]. England, for example, implemented the campaign “Smokefree 2030” in 2019, so interest might have been exhausted [62]. Perhaps as a consequence of the campaign or the pandemic, England saw a diminishing number of smokers in 2020 [63]. Furthermore, less social interaction and heightened awareness of severe health risks might have resulted in the coincidence of fewer new smokers during the pandemic. Notably, studies regarding the initiation of smoking habits during the pandemic are missing. Regular users even with increased consumption might not significantly contribute to search volume because of satisfied informational demand. Matching this hypothesis, a study from Italy presents a decrease in smoking prevalence while overall cigarette consumption increased, attributed to regular users [64].

Second, there is a methodological shortcoming of RSV data. These data are a relative indicator of interest compared with general interest in all searched keywords. As other keywords rise, the shares of interest for the investigated keywords decline, even if the absolute number of search queries remains stable. Especially during the COVID-19 pandemic, this might introduce a bias as COVID-19–related keywords rose exponentially [46]. To account for this shortcoming, we used a moving median approach in our analysis as laid out in the Methods section. This way, clusters were evaluated relative to a period of 1 year, and long-term trends were less influential on cluster evaluation. Accordingly, when looking into cluster accumulation during COVID-19 pandemic, stable numbers were observed as opposed to the comparison of plain RSV.

In the context of cluster detection, attribution to events beyond the scope of this study must be considered. Here, 2 clusters are striking. In Germany, interest in “Tabak” peaked from April to July 2020, which was most likely caused by COVID-19–related import restrictions. Cheap imported cigarettes were not available, so customers shifted their consumption behavior [41]. Also, the pattern of repetitive short-lasting interest in cigar at the time of the start of the vaccination campaign should be carefully interpreted as this might coincide with behavior during festivities. Still, it sticks out that during the pandemic, these clusters were absent. We hypothesize that due to reduced social gatherings around the holidays, people shifted their interest, and especially occasional cigar consumption was thus limited.

### Cessation

Interest for cessation leveled during the pandemic across all countries, seen in overall RSV and number of clusters. Specific keywords where significance was missed should be interpreted with caution due to high variability in search volume. Regarding cessation during the pandemic, most identified survey studies point toward increased interest in cessation, mainly due to a fear of the disease [20,21,24,65]. However, these studies are frequently victim to effects like social desirability [66]. Also, cohort selections mark a large confounder.

Studies on RSV data are in line with our findings and indicate constant or decline in cessation interest [16,19,36]. However, 2 of these investigations solely entailed a visual assessment of the Google search data without using statistical methodologies. Few keywords were analyzed, and the scope was limited to either a single country or the global RSV data. This approach, given the globally diverse trajectory of the pandemic, appears lacking in specificity. On the contrary, the presented approach provides more objective data analysis. Furthermore, recent publications indicate that an increased level of stress hinders smokers from abstinence, and cessation programs switched to disadvantageous remote settings or were discontinued [20,22,56,57,64].

### Treatment

For treatment, we saw diverse developments. There was a decline in e-cigarettes throughout. This trend was evident in other studies, especially in a young population and at the beginning of the pandemic [67-69]. However, a cross-sectional study by Gallus et al [70] reveals that the effect is probably cohort-dependent. Mostly, adolescents decreased consumption, attributed to harder access because of fewer social gatherings [70,71]. In line with this, we observed a lasting cluster for “sigaretta elettronica” in Italy from January to March 2021, and during this time, the incidence declined and restrictions were less harsh, implying an increase in social gatherings. The cluster showed an abrupt end with the start of the third lockdown [72]. RSV data are anonymized, and no conclusion about subgroups can be drawn. Previous studies elaborated on the disparities of internet use for health-related topics in different age groups [73,74]. As this is a limitation of the method that might account for further differences between the presented results and results from cohort studies. Hence, studies investigating internet use for cessation-related topics among different age groups are needed.

The categorization of e-cigarettes within the category of treatment is debatable. In some instances, e-cigarettes solely serve recreational purposes, particularly among the younger demographic [75,76]. The analysis of RSV revealed a notable similarity between e-cigarettes and keywords of consumption, with a decline in both cases. The classification of e-cigarettes within the treatment category stems from comprehensive meta-analyses, demonstrating their efficacy as treatment products [39]. Furthermore, the National Health Service recommendations explicitly advise against the use of e-cigarettes by nonsmokers [77]. Motivational factors influencing e-cigarette usage have been investigated, with prevailing evidence suggesting a large proportion of users use e-cigarettes as aids for smoking cessation or reduction [75,78,79]. Especially, these goal-oriented users continued consumption [80]. Consequently, the categorization of e-cigarettes within treatment aligns with the observed patterns described in previous studies.

Temporal analysis revealed a peaking interest in “Champix” in Spain starting before the pandemic. This is most likely confounded by the coverage of Champix cost by the Spanish health insurance at the beginning of 2020 [81]. Another season-dependent effect was seen in England, Italy, and Spain, with repetitive clusters for treatment around New Year before the pandemic but not during the pandemic. We argue that people shifted their New Year’s resolutions during the health crisis.

### Limitations

Alongside the previously mentioned shortcomings of RSV data, further limitations shall be discussed. First, changes in lockdown policies were passed by the day and hence any cluster detected during the pandemic could have been attributed to one of these changes or diverse non–COVID-19-related occurrences. However, to ensure compatibility across countries, we had to limit the investigated events to major lockdowns, rises in incidence, and vaccinations as we anticipated these would create drastic changes in RSV.

Second, keyword selection poses a bottleneck for Google Trends studies as the selection is mostly reliant on expert consensus, and literature review and is limited by the data provided by Google Trends. Here, relevant terms might be missed or might be searched in a divergent context. By choosing the reference terms based on criteria described in the *Methods* section with only minor changes between countries introduced by translation or availability of keywords provided by Google Trends, we aimed to be as objective as possible. Furthermore, 3 groups of consumption, cessation, and treatment display various aspects of smoking behavior and 3 reference terms per group should suffice to mirror products and word usage prevalent in society.

Third, only 1 search engine and hereby internet-based source was used for data acquisition. Although Google dominates the market concerning search engines, further research might encompass other valuable internet-based resources, such as social media platforms and news forums, to estimate population response to public health crises [82-84].

Furthermore, Google Trends data retrieved for an entire country are low in regional resolution, especially under consideration of disparate incidence rates in national regions information might be lost.

### Implications of the Presented Results

For optimal allocation of resources, it is crucial to evaluate the impact of a health crisis on a vulnerable population. During the COVID-19 pandemic, smokers were among this population [7]. The optimal timing of public health intervention often remains elusive. Survey studies in this setting are disadvantageous. Google Trends studies, in contrast, mirror real-time effects, investigate a large share of the population, and are cost effective. By screening a plethora of keywords, appropriate interventions might be determined. Here, Ayers et al [85] demonstrated an interesting approach to identify search terms and behavioral shifts upon tax increases on cigarettes.

In this study, the decline in cessation interest could have justified policy makers’ efforts to intensify campaigns informing about cessation programs during the pandemic. A more detailed analysis of terms associated with cessation, such as quitlines and local support groups, would be required in further studies to filter optimal interventions. Besides, interest in treatment options seemed to stagnate. Here health care providers could have increased their efforts to educate people about therapy strategies.

Finally, RSV data have provided useful insights for various research in medicine [16,26,28-30,32,36]. Future studies in the context of smoking and health crises could use these data to predict smoking trends early to expand cessation programs and also to monitor the potentially unexpected detrimental effects of other public health measures on smokers. However, there is a need for the following studies to develop methods to deal with shortcomings in keyword selection, regional resolution, and subgroup analysis.

### Conclusion

Trends were comparable across all countries with minor differences. A decline in interest in consumption and cessation was observed. Besides, treatment terms showed heterogeneous dynamics, while specifically, e-cigarettes displayed a markedly decreased RSV. Temporal clusters of peaked RSV were only sparsely linked to lockdown measures and changes in incidence. The flexible scan statistics proved as a valid tool for cluster detection. The resulting clusters corresponded with visual inspection and could partially be linked to events other than lockdown measures or vaccination campaigns. This study underlines the importance of intensifying cessation aid considering the decreased interest during the pandemic. Measures that positively affect smoking behavior in times of health crisis remain to be determined.

## Appendix

Multimedia Appendix 1 Exemplary screenshots of relative search volume (RSV) data from Google Trends for German search terms commonly linked to the pandemic. Starting in the week of the first reported case in Europe, there is an emerging interest in the indicated terms.

## Abbreviations

RSV: relative search volume
WHO: World Health Organization
