# Supplementary figures and images for: Google Trends Assessment of Keywords Related to Smoking and Smoking Cessation During the COVID-19 Pandemic in 4 European Countries: Retrospective Analysis
Source: Online J Public Health Inform. 2024 Dec 3;16:e57718. doi: 10.2196/57718 (PMC11653046; doi:10.2196/57718)

**Figure S1**


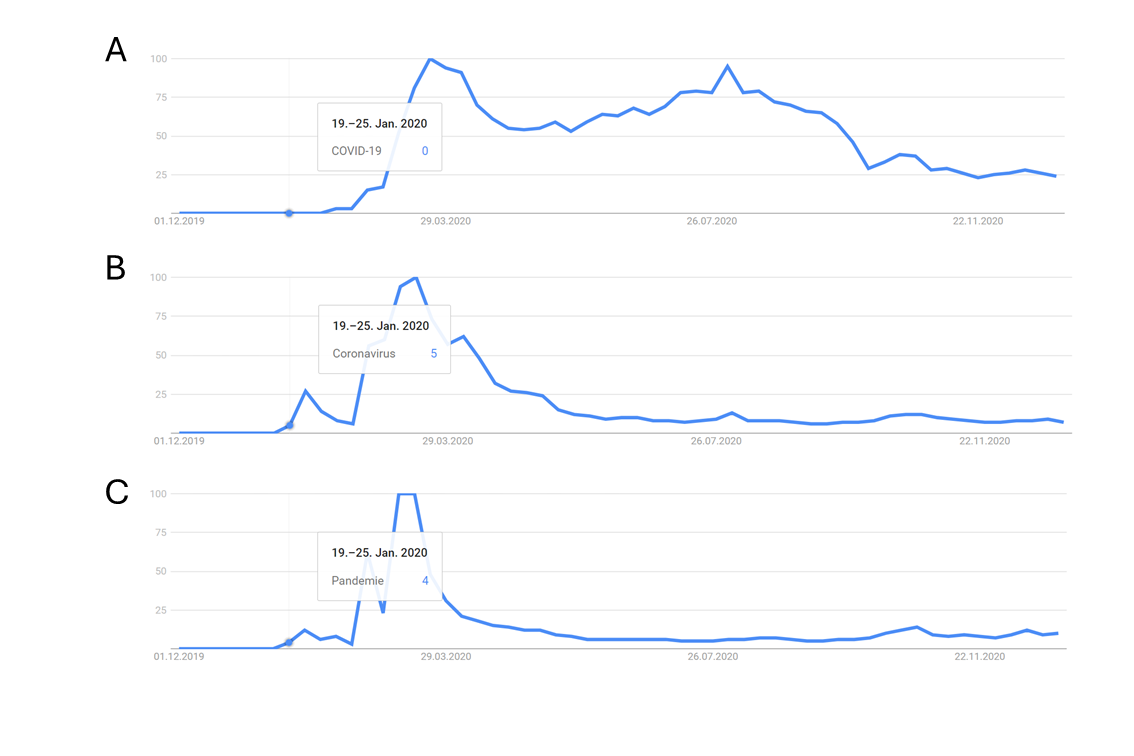

Supplement: Multimedia Appendix 1 [file ojphi_v16i1e57718_app1.docx]
